# Cross-plane shear wave elastography for viscoelasticity imaging

**DOI:** 10.1088/1361-6560/ae7eea

**Published:** 2026-07-02

**Authors:** Ryan P Pitsinger, Murthy N Guddati

**Affiliations:** Department of Civil, Construction, and Environmental Engineering North Carolina State University, Raleigh, NC, United States of America

**Keywords:** shear wave elastography, full waveform inversion, cross-plane imaging, regularization, viscosity imaging

## Abstract

*Objective.* Shear wave elastography (SWE) is widely used for elasticity imaging, but conventional implementations remain confined to two-dimensional (2D) measurement planes and lack sensitivity to viscosity, an emerging biomarker linking shear wave dissipation and disease progression. The objective of this work is to extend SWE for viscoelasticity imaging beyond the measurement plane through methodological and acquisition-based advances. *Approach.* We introduce a cross-plane acquisition strategy in which multiple measurement planes are recorded per acoustic radiation force push to capture dissipation across planes. We perform imaging using recently developed full-waveform inversion, enhanced with an $H^1$ regularization scheme that stabilizes reconstructions by penalizing spurious oscillations. The method is validated using synthetic datasets generated using a reduced-dimension proof-of-concept model focused on out-of-plane sensitivity under varying inclusion geometries, contrasts, and noise levels. Reconstructions were performed using a multiresolution sequential inversion framework that progressively refines elasticity ($G$) and viscosity ($\eta$). *Main results.* The cross-plane strategy significantly improved the recovery of viscosity distributions by capturing dissipative behavior between measurement planes, while $H^1$ regularization enhanced stability and suppressed noise-induced artifacts without oversmoothing. Elasticity maps remained consistent across all cases, whereas viscosity reconstructions exhibited improved boundary fidelity and reduced ambiguity compared with single-plane configurations. *Significance.* These findings demonstrate the feasibility of reconstructing viscoelastic properties beyond the measurement plane using cross-plane SWE. The proposed framework establishes a pathway toward true volumetric (3D) viscoelastic imaging using standard 2D ultrasound acquisitions.

## 1. Introduction

### 1.1. Background and motivation

Ultrasound shear wave elastography (SWE) enables quantitative elasticity imaging by tracking shear wave propagation generated by acoustic radiation force (ARF) excitations (Greenleaf *et al* 2003, Palmeri and Nightingale 2011). The measured wave speed is directly related to tissue stiffness, allowing the construction of elasticity maps that serve as biomarkers for diseases such as liver fibrosis and breast cancer (Barr *et al* 2015, Dietrich *et al* 2017). However, biological tissue is not purely elastic; it exhibits viscoelastic behavior, characterized by both instantaneous (elastic) and time-dependent (viscous) responses (Fung 1993). Viscosity has been linked to pathological processes including tumor progression and liver disease (Ormachea and Parker 2020), suggesting that joint reconstruction of elasticity and viscosity could yield more specific diagnostic insight (Kumar *et al* 2018). Yet, viscosity is challenging to image because its effect manifests primarily through wave attenuation, leading to reduced amplitude, low SNR and thus poor images (Chen *et al* 2009, Kazemirad *et al* 2016, Elmeliegy and Guddati 2024).

Most ultrasound elastography techniques reconstruct parameters only within the two-dimensional (2D) measurement plane (Palmeri and Nightingale 2011, Doyley 2012, Osika and Kijanka 2024). Within these planes, dispersion-based and attenuation-based SWE methods recover viscosity by fitting an assumed rheological model to the wave-speed-versus-frequency curve (Chen *et al* 2009, Deffieux *et al* 2009) and the spatial decay of wave amplitude (Bernard *et al* 2017, Nenadic *et al* 2017), respectively, but both rely on simplified wave-propagation models, which limits their accuracy in heterogeneous tissue. Local phase velocity imaging (Kijanka and Urban 2021), viscoelastic-response (VisR) imaging (Hossain and Gallippi 2020), and frequency-shift viscosity reconstruction (Bhatt *et al* 2019) are imaging methods that produce viscoelastic maps but are confined to the 2D plane. Beyond the 2D plane, approaches for 3D SWE include external-vibration acquisition on conventional scanners (Huang *et al* 2020), 4D ultrafast volumetric imaging with matrix-array probes (Tanter and Fink 2014, Gennisson *et al* 2015), and system-identification voxel-wise estimation of viscoelastic parameters (Li *et al* 2025). These methods either omit viscosity or treat it through a 1D wave model, which limits the accuracy of spatial viscoelastic mapping. None employ full waveform inversion (FWI), a method originally developed in geophysics (Tarantola 1984, Virieux and Operto 2009). Recent work has demonstrated that FWI can be adapted to elastography to recover viscoelastic properties beyond the measurement plane (Elmeliegy and Guddati 2024). These studies illustrated that elasticity can be reconstructed successfully, including regions outside the measurement plane. For viscosity, however, the accuracy of reconstructions drops significantly away from the measurement plane and is generally limited to regions near the plane. This is because wave motion and energy loss due to viscosity are more difficult to resolve out of the plane (Elmeliegy and Guddati 2024).

Motivated by these findings, the present study extends viscoelastic FWI with two major innovations. First, we introduce an $H^1$ regularization scheme that stabilizes reconstructions by penalizing high spatial gradients, suppressing noise-induced oscillations. Second, we propose a cross-plane imaging strategy in which multiple measurement planes are recorded per ARF push, rather than a single plane. This addition explicitly captures how energy dissipates between planes, enhancing the conditioning of the viscosity inversion and improving recovery of out-of-plane features. To isolate and study this behavior, we use a reduced-dimension configuration where the ARF and inclusions are assumed invariant in the axial ($z$) direction, collapsing the measurement to a line in $x$ but reconstructing in the $x$–$y$ plane. This model captures the essential physics of out-of-plane viscoelastic imaging for this proof-of-concept study while maintaining an acceptable computational burden.

By combining multi-scale correlation-based FWI with cross-plane acquisition and $H^1$ regularization, we demonstrate significant improvements in the stability and fidelity of viscosity reconstructions. Using synthetic datasets with controlled inclusions and varying noise levels, the results highlight the benefits of cross-plane sampling for capturing dissipative wave behavior. To our knowledge, this represents the first demonstration of cross-plane SWE for ultrasound elastography, establishing a foundation for future three-dimensional (3D) viscoelastic imaging from conventional 2D measurements.

## 2. Methods

The proposed methodology extends FWI for viscoelastic imaging in SWE by introducing a cross-plane acquisition strategy and $H^1$ regularization to improve the stability and accuracy of viscosity reconstructions. This section details the problem setup, the governing physics, the FWI framework, and the specific enhancements developed in this work.

### 2.1. Problem setup

**Figure 1.**
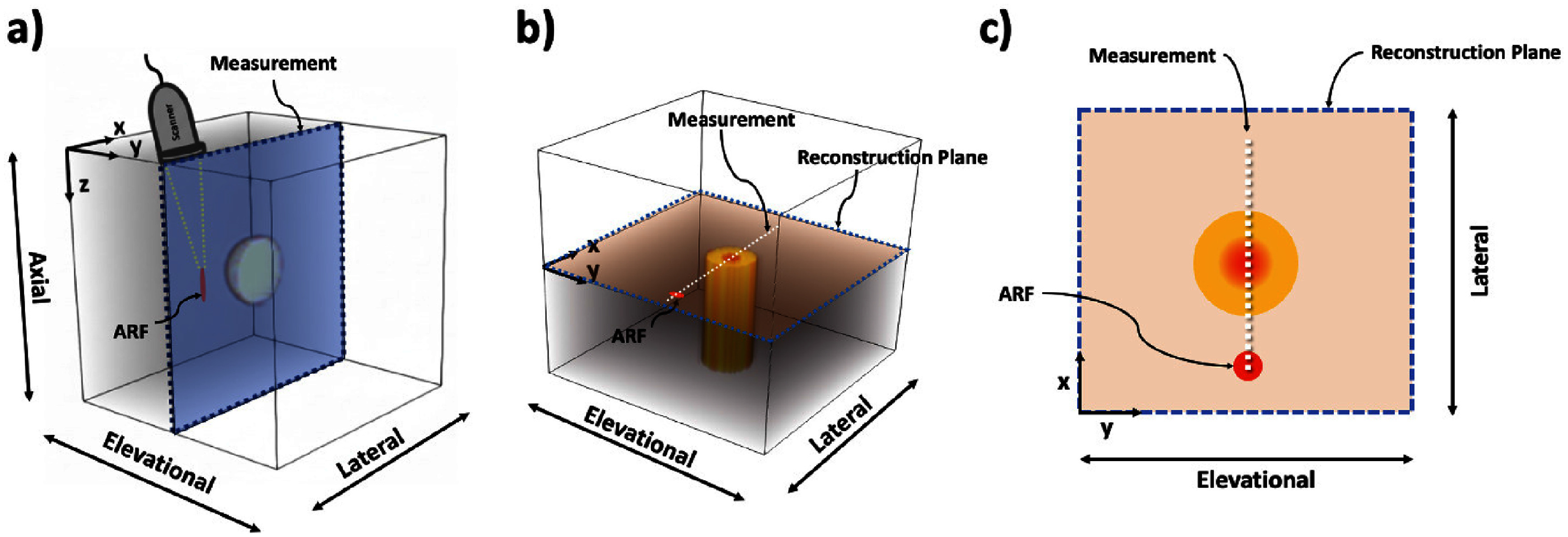
Schematic of the problem setup. (a) Conventional SWE with a 3D inclusion and ARF, where measurements are restricted to 2D planes. This represents the practical challenge of using 2D data to reconstruct fully 3D structures. (b) Reduced model problem adopted in this work, where both the ARF and inclusion are assumed invariant along the axial ($z$) direction. In this setting, the out-of-plane challenge translates to reconstructing a 2D $x$–$y$ slice from 1D line measurements. (c) Equivalent 2D schematic of (b), showing the reconstruction restricted to the $x$–$y$ plane while retaining the essential out-of-plane imaging challenge.

In SWE, an ARF generates shear waves, and the resulting wave speed is used to infer tissue stiffness. While the imaging domain is inherently three-dimensional, conventional SWE measurements are typically confined to a 2D slice (figure 1(a)), limiting sensitivity to out-of-plane structures and viscous properties (Elmeliegy and Guddati 2023, 2024).

To establish a tractable proof of concept for out-of-plane imaging, we consider a reduced-dimension model where both the ARF excitation and the tissue inclusions are assumed to be invariant along the axial ($z$) direction (figure 1(b)) (Elmeliegy and Guddati 2024). This simplification reduces the problem to a 2D reconstruction in the $x$–$y$ plane from measurements along a line in the $x$ direction (figure 1(c)), preserving the essential physics of viscoelastic wave propagation while isolating the core challenge of out-of-plane imaging. In making this simplification, we are reducing the effect of geometric spreading and altering the effect of out-of-plane scattering, with the former adding to the true attenuation in actual 3D SWE. A 3D extension to more fully capture these mechanisms would require a 3D forward model and 2D measurement planes (figure 1(a)). This reduced-dimension setup is analogous to geophysical imaging, where surface or line measurements are inverted for an image through the depth (Tarantola 1984, Virieux and Operto 2009), motivating the use of inversion frameworks that exploit full-wave physics rather than relying solely on time-of-flight analysis.

### 2.2. FWI framework

The viscoelastic parameters were reconstructed using an FWI framework designed to minimize the misfit between measured and simulated particle displacements by iteratively updating the material model (Tarantola 1984, Virieux and Operto 2009). This framework builds upon prior work (Elmeliegy and Guddati 2024), for which we restate the key contributions and describe the improvements.

**Objective function:** The misfit was quantified using a correlation-based objective function, which is less sensitive to unknown ARF parameters such as amplitude and profile compared to traditional least-squares methods (Elmeliegy and Guddati 2023, Roy and Guddati 2023). They have also been observed in FWI applications to yield a wider basin of attraction and greater robustness to noise (Elmeliegy and Guddati 2023). The baseline objective was defined as

\begin{equation*} J_0\left(p\right) = 1 - \frac{\left| d \cdot \overline{u}\left(p\right) \right|^2}{\|d\|^2 \, \|\overline{u}\left(p\right)\|^2},\end{equation*}

where $d$ is the measured displacement field and $\overline{u}(p)$ denotes the frequency-domain simulated displacement for parameters $p$ (the overbar denotes a frequency-domain quantity throughout). Importantly, the objective function is evaluated only at the 1D line measurement locations, rather than over an entire 2D field. Thus, the inversion compares simulated and measured displacements only along these sampled receiver lines (illustrated schematically in figure 1(c)). In this reduced setting, 1D measurements are used to reconstruct 2D viscoelastic maps, providing a proof-of-concept for the more general problem of using 2D planar measurements to reconstruct 3D viscoelasticity in practice. A value of $J_0(p) = 0$ signifies a perfect match (Elmeliegy and Guddati 2023). Gradient computation of the baseline objective function (1) is detailed in the following section.

**Gradient computation via adjoint-state method:** Gradients of the baseline correlation-based objective $J_0(p)$ (1) were computed using the adjoint-state method, a standard approach for PDE-constrained optimization in FWI (Virieux and Operto 2009).

The total derivative of $J$ with respect to the model parameters $p$ can be expressed as

\begin{equation*} \frac{dJ\left(p\right)}{dp} = \frac{\partial J}{\partial p} + \frac{\partial J}{\partial \overline{u}} \frac{\partial \overline{u}}{\partial p},\end{equation*}

where $\overline{u}$ is the displacement field that satisfies the PDE constraint $\mathcal{R}(\overline{u},p) = 0$. Differentiating this constraint gives

\begin{equation*} \frac{\partial \mathcal{R}}{\partial p} + \frac{\partial \mathcal{R}}{\partial \overline{u}} \frac{\partial \overline{u}}{\partial p} = 0,\end{equation*}

leading to

\begin{equation*} \frac{\partial \overline{u}}{\partial p} = -\left(\frac{\partial \mathcal{R}}{\partial \overline{u}}\right)^{-1} \frac{\partial \mathcal{R}}{\partial p}.\end{equation*}

Substituting (4) into (2) yields

\begin{equation*} \frac{dJ\left(p\right)}{dp} = -\frac{\partial J}{\partial \overline{u}} \left(\frac{\partial \mathcal{R}}{\partial \overline{u}}\right)^{-1} \frac{\partial \mathcal{R}}{\partial p}.\end{equation*}

Defining the adjoint variable $\lambda$ as the solution of

\begin{equation*} \left(\frac{\partial \mathcal{R}}{\partial \overline{u}}\right)^T \lambda = -\left(\frac{\partial J}{\partial \overline{u}}\right)^T,\end{equation*}

the gradient in (5) can be written as

\begin{equation*} g\left(p\right) = \lambda^T \frac{\partial \mathcal{R}\left(\overline{u},p\right)}{\partial p}.\end{equation*}

This formulation avoids explicit differentiation of $\overline{u}$ with respect to $p$, making gradient computation efficient even for imaging problems with a large number of parameters (Plessix 2006).

This compact expression (7) is general and applies to any PDE-constrained optimization. In a subsequent subsection, we specialize this framework to the shear-wave forward model (14) and the correlation-based objective $J_3(p)$ (13), deriving explicit gradients with respect to elasticity and viscosity.

**Forward model:** The forward problem, which simulates shear wave propagation for a given set of material parameters, is governed by the antiplane shear wave equation in the frequency domain (Elmeliegy and Guddati 2024):

\begin{equation*} -\nabla \cdot \left(G^*\nabla\overline{u}\right) - \rho\omega^2\overline{u} = \widehat{ARF},\end{equation*}

where $\overline{u}$ is the frequency-domain displacement, $\rho$ is density, $\omega$ is the angular frequency, and $\widehat{ARF}$ is the external force. The tissue is modeled as a linear viscoelastic material using the Kelvin–Voigt model, which provides a simple representation of frequency-dependent viscoelastic behavior (Catheline *et al* 2004, Chen *et al* 2004). The complex shear modulus is then defined as

\begin{equation*} G^* = G + i\omega\eta,\end{equation*}

where $G(x,y)$ is the shear modulus (elasticity) and $\eta(x,y)$ is the shear viscosity (Elmeliegy and Guddati 2024). To enforce positivity, we use a logarithmic parameterization $p = \{\log(G), \log(\eta)\}$ (Elmeliegy and Guddati 2024). The forward model was solved using the finite element method (FEM) implemented in MOOSE (Permann *et al* 2020), consistent with the reduced problem setup shown in figures 1(b) and (c), where axial invariance is assumed and reconstructions are carried out in the $x$–$y$ plane.

To mimic an infinite domain and suppress spurious reflections from computational boundaries, absorbing boundary conditions were applied. These can be formally expressed as

\begin{equation*} G^* \nabla \overline{u} \cdot \mathbf{n} = \Lambda \overline{u},\end{equation*}

where $\mathbf{n}$ is the outward unit normal and $\Lambda$ is the Dirichlet-to-Neumann operator representing the impedance of the exterior boundary. While there exist more sophisticated versions of $\Lambda$ (Lindman 1975, Engquist and Majda 1977, Higdon 1986, Guddati 2006), we use the simple Sommerfeld boundary condition with $\Lambda = -i\omega\sqrt{\rho G}$ in this study (Elmeliegy and Guddati 2024).

**Inversion scheme:** To enhance stability and improve reconstruction quality, we employed three established strategies from geophysical inversion and other previous work:

1. *Multiresolution strategy:* A key challenge in FWI is cycle skipping, where the inversion incorrectly aligns measured and simulated waveforms to the wrong oscillation period, leading to spurious minima in the objective function (Elmeliegy and Guddati 2023). To mitigate this, reconstructions progressed from coarse to fine spatial discretizations and from low to high frequencies. In practice, this means beginning with a coarse mesh and low-frequency content, then progressively refining the mesh and expanding the frequency band. By starting from smooth, low-frequency content, large-scale features are captured first, ensuring proper waveform alignment before resolving finer details. As higher frequencies are progressively incorporated, the objective function $J_0(p)$ is enriched with additional frequency content, while mesh refinement increases the parameterization, allowing the reconstruction to capture finer spatial features at each stage (Elmeliegy and Guddati 2024).
2. *Sequential inversion:* While elasticity reconstructions converge well due to their strong control over wave kinematics, viscosity remains challenging to recover accurately when both parameters are updated simultaneously throughout the entire inversion. To address this, both elasticity and viscosity were initially updated together until elasticity sufficiently converged. Once the wave kinematics were properly captured, elasticity was held fixed while viscosity was further refined, through reinitializing and optimizing. This staged process ensures that the propagation characteristics are correctly established before isolating dissipative effects, mitigating parameter cross-talk where errors in elasticity can leak into viscosity reconstructions (Elmeliegy and Guddati 2024). Like multiresolution, this does not alter $J_0(p)$ but modifies the update pathway.
3. *Multi-acquisition strategy:* Viscosity effects manifest as dissipation rather than propagation, making them challenging to capture with a single ARF excitation (Prieux *et al* 2013, Budelli *et al* 2017, Elmeliegy and Guddati 2024). To address this, multiple pushes were applied at different spatial locations. The displacement fields from these pushes were combined in a single inversion, which modifies $J_0(p)$ (1) to sum the misfit across pushes:
  \begin{equation*} J_1\left(p\right) = \sum_{i = 1}^{N_s} \left[ 1 - \frac{|d_i \cdot \overline{u}_i\left(p\right)|^2}{\|d_i\|^2 \, \|\overline{u}_i\left(p\right)\|^2} \right],\end{equation*}
  where $N_s$ is the number of ARF pushes. Diverse pushes increase the illumination in the presence of dissipation, improving sensitivity and enhancing reconstruction quality.

**Figure 2.**
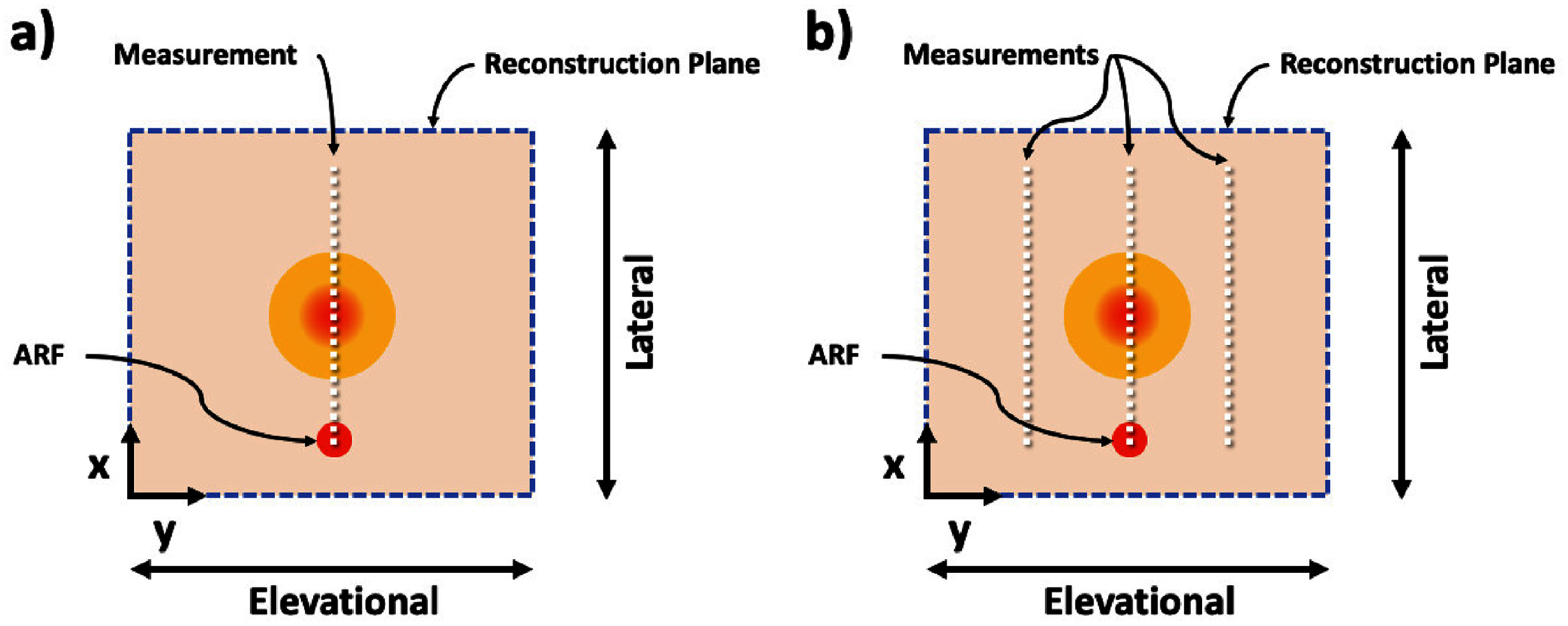
Illustration of inversion strategies. (a) Single-plane measurement per ARF push, which provides limited sensitivity to viscosity. (b) Cross-plane strategy with multiple measurement planes per push, enabling dissipation to be captured across planes and improving viscosity reconstructions.

Despite these strategies, several limitations remain in the current FWI framework. First, single-plane measurement configurations (see figure 2(a)) provide insufficient information to robustly capture viscous dissipation. As demonstrated in prior work (Elmeliegy and Guddati 2024), viscosity can be reasonably reconstructed near the measurement plane, but remains less accurate than elasticity, particularly in regions away from the plane. Without reflectors, single-plane acquisitions fail to adequately sample dissipative behavior along the source–receiver path. We hypothesize that by incorporating cross-plane measurements (figure 2(b)), one can explicitly capture how wave energy dissipates between planes, thereby improving sensitivity to viscosity and reducing the ambiguity inherent in single-plane reconstructions. Second, while multiresolution and sequential inversion improve stability, they do not fully suppress noise-induced oscillations. These limitations motivate the methodological enhancements introduced next, which explicitly address the sensitivity to viscosity and the stability of the inversion process.

### 2.3. Methodological enhancements

To overcome the limitations listed above, we introduced two key enhancements to the FWI framework: a cross-plane acquisition strategy to increase sensitivity to viscosity, and an $H^1$ regularization scheme to improve stability.

**Cross-plane acquisition strategy:** Conventional SWE acquisitions use a single measurement plane per ARF push, providing limited information to quantify viscous dissipation, a known limitation in clinical SWE (Doyley 2012, Kazemirad *et al* 2016). The proposed approach records data on multiple measurement planes for each push (figure 2(b)). This modifies the multi-acquisition objective $J_1(p)$ (11) by extending the summation over measurement planes as well:

\begin{equation*} J_2\left(p\right) = \sum_{i = 1}^{N_s} \sum_{j = 1}^{N_m} \left[ 1 - \frac{\left| d_{ij} \cdot \overline{u}_{ij}\left(p\right) \right|^2}{\|d_{ij}\|^2 \, \|\overline{u}_{ij}\left(p\right)\|^2} \right],\end{equation*}

where $N_m$ is the number of measurement planes per push. This explicitly captures how wave energy dissipates between planes, directly enhancing sensitivity to viscosity (Roy and Guddati 2023) and improving reconstruction accuracy through the correlation-based framework (Elmeliegy and Guddati 2023).

**$H^1$ regularization:** Finally, we incorporated an $H^1$ smoothness term into the cross-plane objective $J_2(p)$ (12) to penalize spurious oscillations in the reconstructed parameters, a common issue with inverting noisy data (Doyley 2012, Prieux *et al* 2013):

\begin{equation*} J_3\left(p\right) = J_2\left(p\right) + \frac{\alpha}{2} \int_{\Omega} \left( |\nabla \log G|^2 + |\nabla \log \eta|^2 \right) \mathrm{d}x\mathrm{d}y.\end{equation*}

$\alpha$ is the regularization weight. The additional integral term acts as a gradient penalty: the $|\nabla \log G|^2$ term discourages sharp, oscillatory variations in elasticity reconstructions; the $|\nabla \log \eta|^2$ term enforces smoothness in viscosity reconstructions. Together, these terms suppress spurious oscillations, while preserving essential features such as inclusion shapes (Doyley 2012).

**Adjoint-state gradient.** Building on the general adjoint framework (equations (2)–(7)), we specialize it here to the viscoelastic FWI setting. In particular, we derive explicit gradients of the correlation-based objective with cross-plane data and $H^1$ regularization, $J_3(p)$ (13). For each push $i$, the forward problem is

\begin{equation*} A\left(p\right)\,\overline{u}_{i} = \widehat{ARF}_i,\end{equation*}

with log-parameters $p = \{\log G,\log \eta\}$ and complex modulus $G^* = G+i\omega\eta$. The full solution $\overline{u}_i$ is obtained for each push, and measurements at different planes $j = 1, \ldots, N_m$ are extracted through observation operators $S_{ij}$. The objective $J_3(p)$ (13) is evaluated only at these line-receiver locations.

The adjoint equations then take the form

\begin{equation*} A\left(p\right)^* \overline{\lambda}_{i} = \sum_{j = 1}^{N_m} S_{ij}^\top g_{ij},\end{equation*}

where $g_{ij}$ is the derivative of the correlation misfit for push $i$ and plane $j$, $\overline{\lambda}_{i}$ is the frequency-domain adjoint variable, and the summation combines adjoint sources from all measurement planes for each push.

Finally, inserting these adjoint solutions into the gradient expression yields the parameter sensitivities:

\begin{align*} \frac{\partial J_3}{\partial G} &amp; = -\Re\!\left[\sum_{i = 1}^{N_s} \nabla \overline{u}_{i}:\nabla \overline{\lambda}_{i}\right] - \frac{\alpha}{G}\Delta \log G,\end{align*}

\begin{align*} \frac{\partial J_3}{\partial \eta} &amp; = -\Re\!\left[\sum_{i = 1}^{N_s} i\omega\,\nabla \overline{u}_{i}:\nabla \overline{\lambda}_{i}\right] - \frac{\alpha}{\eta}\Delta \log \eta.\end{align*}

Applying the log-parameterization gives

\begin{equation*} \nabla_p J_3 = \left\{G\,\frac{\partial J_3}{\partial G}, \;\; \eta\,\frac{\partial J_3}{\partial \eta} \right\}.\end{equation*}

The procedure is summarized in algorithm 1, and implemented in the MOOSE optimization module (Prince *et al* 2024), which is built on PETSc/TAO (Balay *et al* 2025).

**Table:** 

**Algorithm 1.** Cross-plane SWE using the objective $J_3$ (13).
**Inputs:** line-measurement data $\{d_{ij}\}$ from $N_s$ pushes and $N_m$ planes; sequence of meshes/frequency bands $\{\mathcal{M}_r\}_{r = 1}^R$ (coarse$\rightarrow$fine, low$\rightarrow$high); initial model $p_0 = \{\log G,\log \eta\}$; parameter bounds and optimization tolerances.
**Objective:** minimize $J_3(p)$ (13) over $\Omega$.
1. **For** resolution level $r = 1,\dots,R$ **do**
(a) Set working mesh/frequency band to $\mathcal{M}_r$; warm start $p_r \leftarrow p_{r-1}$ from previous level.
(b) **Repeat**
i. *Forward solve:* for each push $i = 1,\dots,N_s$, solve equation (14) to obtain the full solution
$\overline{u}_{i}(p)$, then extract measurements at each plane $j = 1,\dots,N_m$ using observation
operators $S_{ij}$.
ii. *Objective:* evaluate $J_3(p)$ using (13).
iii. *Adjoint/gradient:* For each push $i$, solve the adjoint equation (15) combining
contributions from all planes, then compute the parameter gradient $\nabla_p J_3(p)$
using (16–18).
iv. *Bound-constrained (BFGS):* compute a search step $\Delta p$ using limited-memory
inverse-Hessian update with bound handling.
v. *Projected update (unit step):*
$p \leftarrow \mathrm{proj}_{[l,u]}(p + \Delta p).$
vi. *Stopping tests:* stop if any hold:
$\|\nabla_p J_3(p)\|_{\mathrm{rel}} \unicode{x2A7D} \text{relative gradient tolerance}$,
$\|\nabla_p J_3(p)\| \unicode{x2A7D} \text{absolute gradient tolerance}$,
$k \unicode{x2A7E} \text{maximum iterations}.$
(c) **until** a stopping test is satisfied.
2. **Sequential refinement:** fix $G$ at its current estimate and repeat the inner loop at
the finest level updating only $\eta$ (reduces cross-talk).
3. **Output:** reconstructed elasticity $G(x,y)$ and viscosity $\eta(x,y)$ from $p$.

### 2.4. *In silico* validation

**Figure 3.**
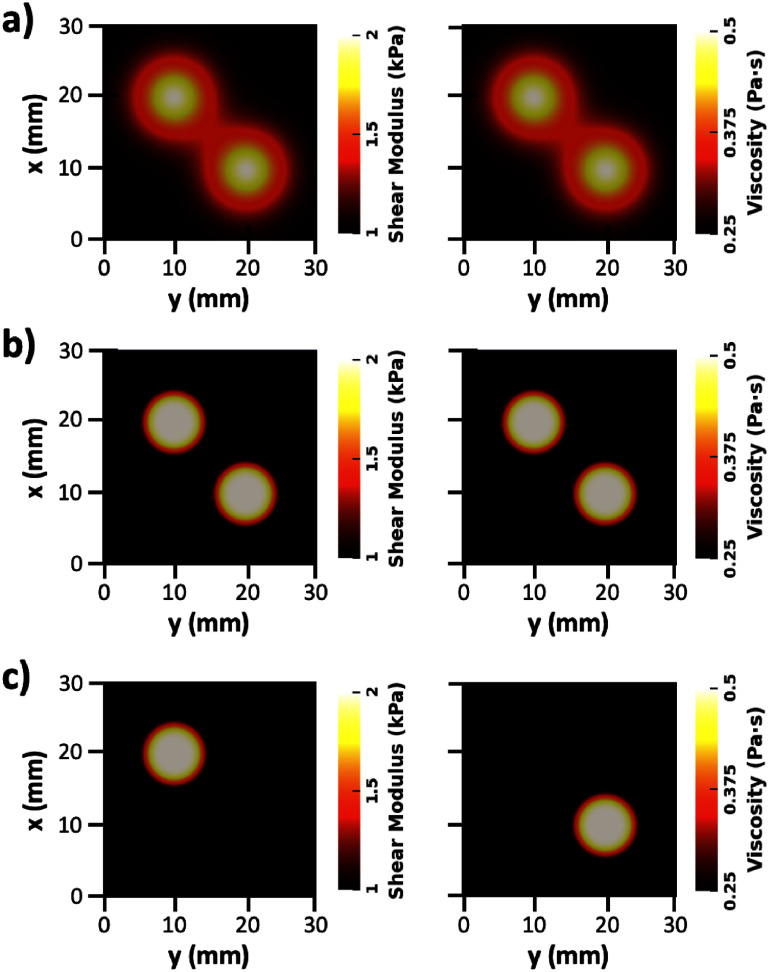
True distributions for the three inclusion types: (a) bimodal Gaussian, (b) sharp-contrast, and (c) uncorrelated. For each case, shear modulus **(left)** and viscosity **(right)** are shown.

All synthetic datasets were generated using 2D FEM simulations on a $60 \times 60$ mesh over a $30 \times 30\ \mathrm{mm}$ region of interest (ROI). Shear wave propagation was modeled at two excitation frequencies (100 and 200 Hz), within the clinically relevant band for SWE (Budelli *et al* 2017, Kijanka and Urban 2021, Lim *et al* 2023). The background material values were set to $G = 1\ \mathrm{kPa}$ and $\eta = 0.25\ \mathrm{Pa \cdot s}$, representative of values reported for soft tissue and tissue-mimicking phantoms (Chen *et al* 2004, Sinkus *et al* 2005), with inclusions defined to introduce localized variations in elasticity and viscosity. Three inclusion types were examined (see figure 3):

a. *Bimodal Gaussian:* Two smooth lobes centered near $(10,20)$ mm and $(20,10)$ mm with widths of approximately 4 mm, producing gradual variations in both $G$ and $\eta$.
b. *Sharp-contrast:* Inclusions at the same locations but with narrower 3 mm profiles and steep boundaries, representing abrupt transitions.
c. *Uncorrelated:* Elasticity and viscosity fields generated independently, such that high-$G$ and high-$\eta$ regions did not coincide.

**Figure 4.**
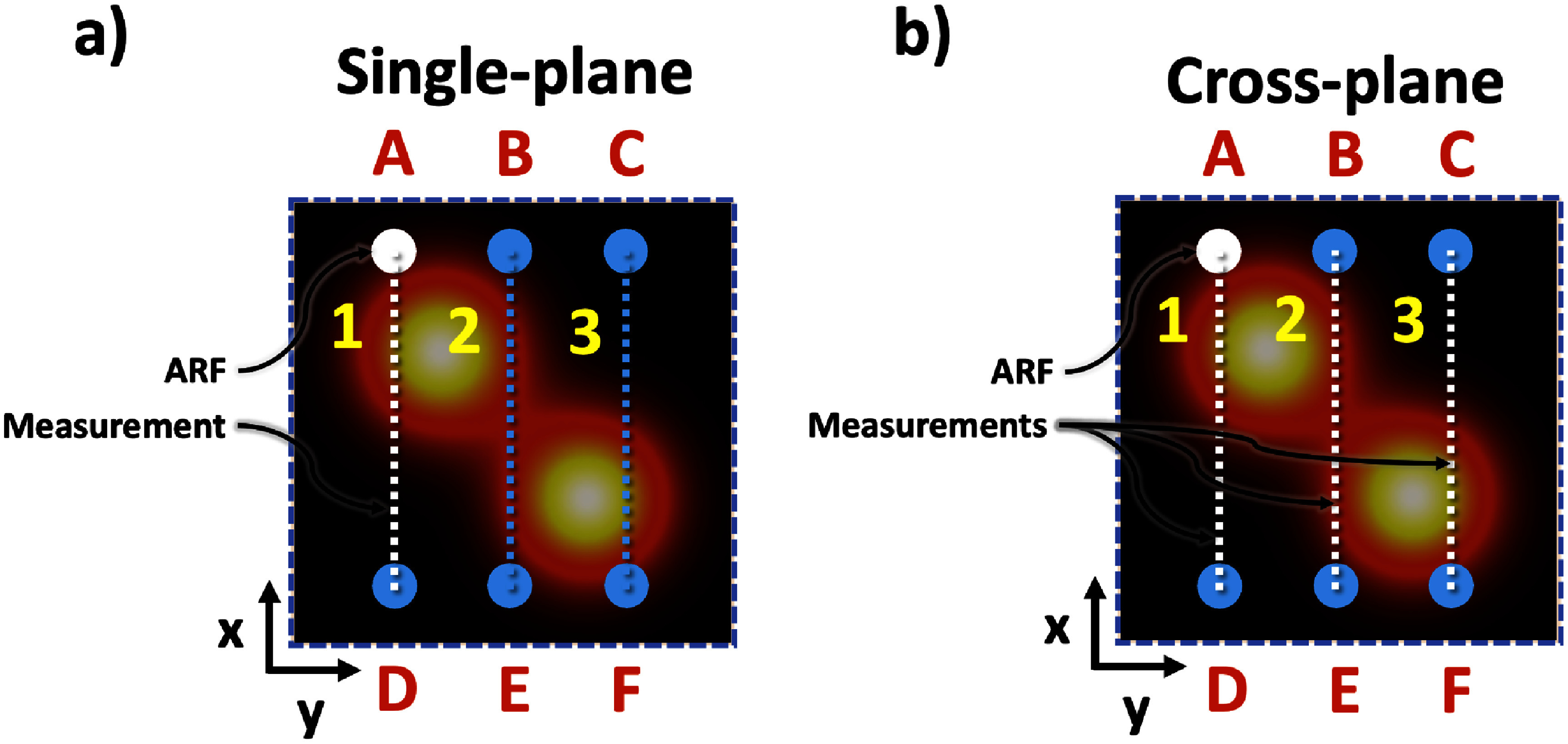
Measurement configurations for single-plane (a) and cross-plane (b) strategies within a $30 \times 30\ \mathrm{mm}$ ROI (see figure 2 for schematic context). Six ARF pushes are used in both configurations, with push locations labeled **A–F** (circles) and measurement lines labeled **1–3** (vertical dashed lines). (a) In the single-plane configuration, each ARF push (A)–(F) is paired with a single measurement line (e.g. A1, B2, C3, D1, E2, F3). (b) In the cross-plane configuration, the same six push locations are combined with multiple parallel measurement lines, allowing each push to acquire data along all three lines (A123, B123, C123, etc). The white-highlighted push and lines indicate one active ARF event and its corresponding measurement geometry.

Reconstructions employed a multiresolution scheme, beginning with coarse meshes ($3 \times 3$, $6 \times 6$) at 100 Hz and progressively refined using both frequencies on finer meshes ($12 \times 12$, $24 \times 24$). At the final resolution, a sequential inversion step was performed in which elasticity ($G$) was fixed and only viscosity ($\eta$) was refined. An $H^1$ gradient penalization term with regularization weight $\alpha = 10^{-3}$ was applied throughout to suppress oscillations while preserving inclusion boundaries. This setup, consistent with the schematic in figure 2, relies on the specific measurement configurations described next (figure 4).

#### 2.4.1. Measurement configurations

The measurement configurations for single-plane and cross-plane setups are shown in figure 4. Six ARF pushes (A–F) were applied in both cases, with lateral positions aligned to the measurement lines at $x = 5$ mm for the bottom row and $x = 25$ mm for the top row of pushes. Each ARF push was modeled as a piecewise linear function with unit amplitude at its center, decaying to zero within approximately 1 mm. This simplified profile approximates the highly localized excitation produced by focused ultrasound transducers (Palmeri *et al* 2017, Lim *et al* 2023, Osika and Kijanka 2024). In the single-plane configuration (figure 4(a)), each push is paired with a single vertical measurement line, representing conventional SWE geometry. In the cross-plane configuration (figure 4(b)), the same push locations are used, but displacement is recorded along three vertical measurement lines at $y = 7$, 15, and 23 mm. Displacement fields were sampled at 0.2 mm intervals along each measurement line over the range $x \in [6, 24]$ mm, within the range of spatial sampling used in SWE simulation literature (Van Sloun *et al* 2017, Osika and Kijanka 2024).

Synthetic data were generated via forward FEM simulations in MOOSE, then corrupted with multiplicative random noise: $u_{\mathrm{noisy}} = u_{\mathrm{true}} \cdot (1 + \epsilon \cdot r)$, where $r \sim U(0,1)$ and $\epsilon = 10^{-\mathrm{SNR}/20}$. Three noise levels were examined: no noise (ideal case), 30 dB SNR, and 15 dB SNR, representing progressively more challenging measurement conditions. A similarly simplified noise model is adopted in the prior correlation-based FWI studies on which the present method directly builds (Elmeliegy and Guddati 2023, 2024), and we keep the treatment at this level of simplification so that the new contributions of cross-plane acquisition and $H^1$ regularization can be assessed against an established baseline rather than confounded with a change in noise treatment. As it is only multiplicative noise, it ignores speckle decorrelation and displacement-tracking jitter (Walker and Trahey 1995), and additive system noise, and validation under more realistic noise is an important direction for future work.

## 3. Results and discussion

### 3.1. Effect of regularization

**Figure 5.**
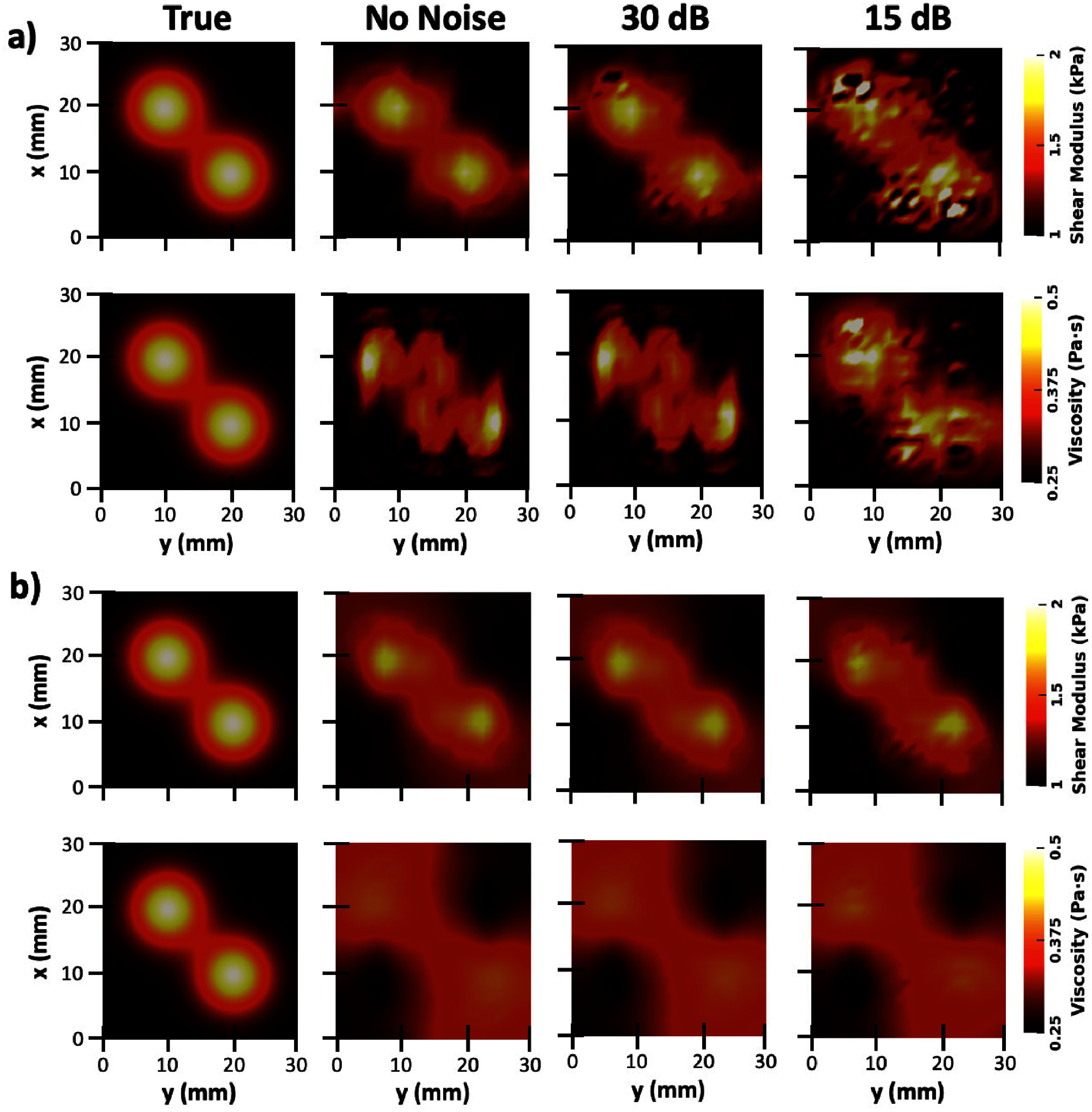
$H^1$ regularization results for the bimodal Gaussian inclusion using six pushes with one measurement line each (push configuration in figure 4(a)). (a) Reconstructions without $H^1$ regularization, corresponding to the objective $J_1(p)$ (11). (b) Reconstructions with $H^1$ regularization, corresponding to the objective $J_1(p)$ (11) now with the regularization term from $J_3(p)$ (13) with weight $\alpha = 10^{-3}$. For each case (a), (b), columns show ground truth, no noise, 30 dB SNR, and 15 dB SNR; top row shows shear modulus maps and bottom row viscosity.

The impact of $H^1$ regularization is illustrated in figure 5 for the bimodal Gaussian inclusion using six ARF pushes under the single-plane configuration (figure 4(a)). Reconstructions without regularization correspond to the objective $J_1(p)$ (11) and exhibit oscillatory artifacts, particularly in the viscosity maps, due to the ill-posed nature of the inverse problem (Doyley 2012). Incorporating $H^1$ regularization, as defined in the objective $J_1(p)$ (11) now with the regularization term from $J_3(p)$ (13) with weight $\alpha = 10^{-3}$, smooths these oscillations while preserving inclusion boundaries and contrast. The improvement is especially evident at lower SNR levels, where the regularization effectively suppresses noise-induced variations without oversmoothing key structural features. The same qualitative behavior was observed for the sharp-contrast and uncorrelated inclusions (not shown for brevity).

### 3.2. Effect of cross-plane strategy

**Figure 6.**
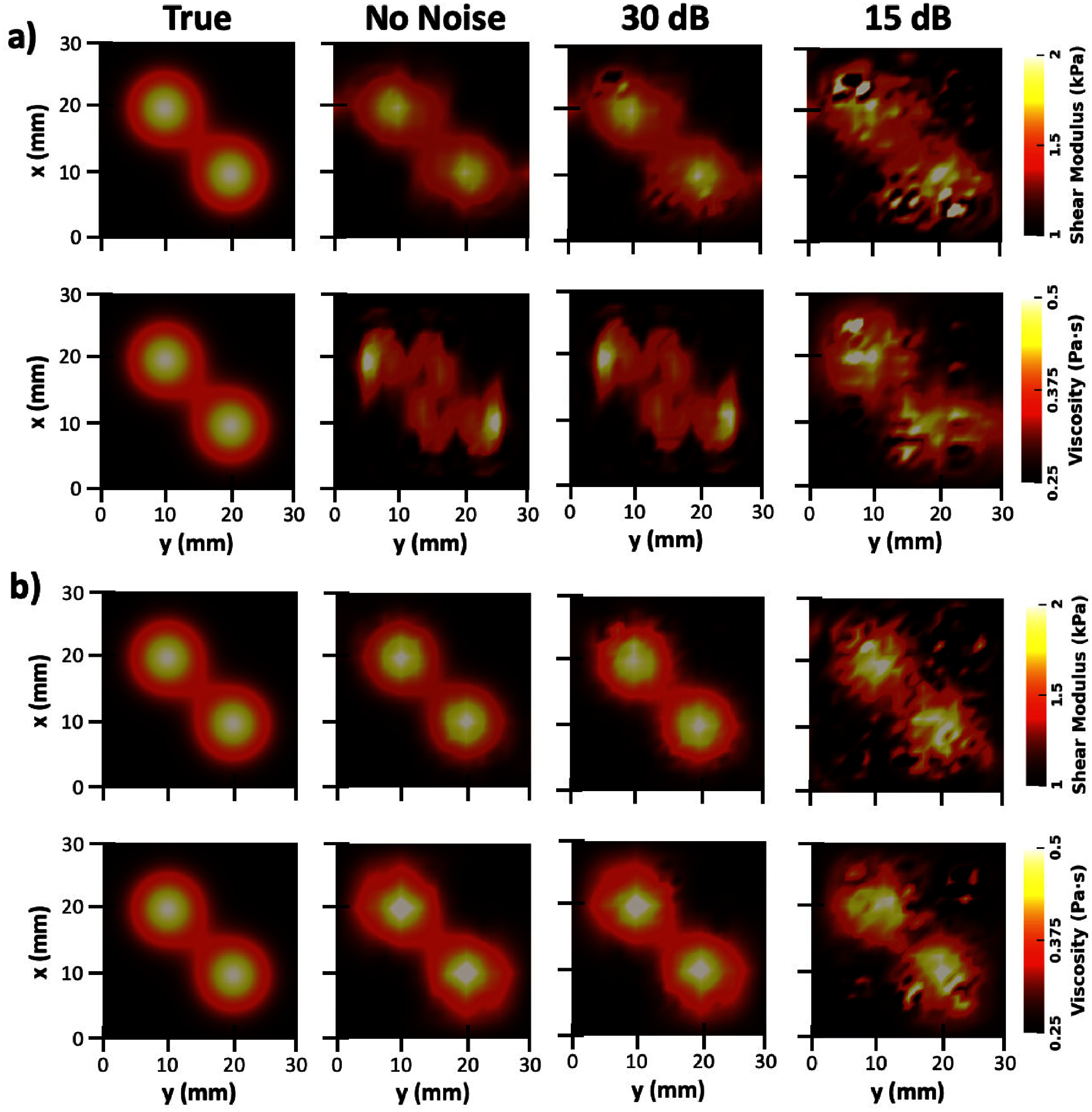
Effect of the cross-plane strategy on reconstructions *without* $H^1$ regularization for the bimodal Gaussian inclusion using six pushes (see configuration in figure 4). (a) Single-plane configuration corresponding to figure 4(a), reconstructed using the objective $J_1(p)$ (11). (b) Cross-plane configuration corresponding to figure 4(b), reconstructed using the extended objective $J_2(p)$ (12). For each case (a) and (b), columns show the ground truth, no noise, 30 dB SNR, and 15 dB SNR; rows show shear modulus (top) and viscosity (bottom).

The effect of cross-plane sampling is demonstrated in figure 6 for the bimodal Gaussian inclusion using six ARF pushes (see configuration in figure 4). Reconstructions without cross-plane measurements correspond to the single-plane objective $J_1(p)$ (11), shown in figure 4(a), while those with cross-plane measurements correspond to the extended objective $J_2(p)$ (12), shown in figure 4(b). Increasing the number of measurement planes from one to three markedly improves the recovery of both shear modulus and viscosity distributions, particularly under noisy conditions. The additional measurement planes capture the energy dissipation between planes, improving sensitivity to viscosity and reducing ambiguity caused by limited single-plane coverage. Quantitatively, the cross-plane reconstructions shown in figure 6 reduce the RMS error by 58% for viscosity and 62% for elasticity at 30 dB SNR (59% and 67% respectively at no noise; 22% and 49% at 15 dB). The cross-plane improvement was likewise observed for the sharp-contrast and uncorrelated inclusions (not shown for brevity).

### Combined effect of cross-plane acquisition and $H^1$ regularization

3.3.

**Figure 7.**
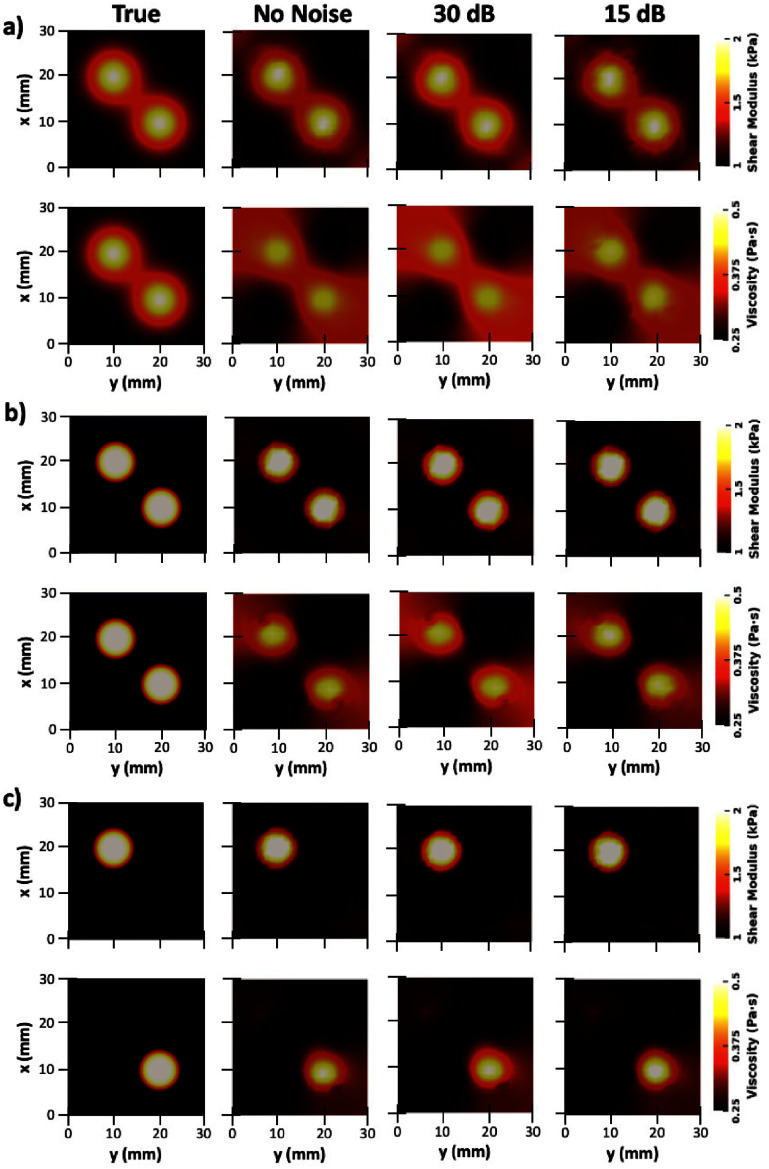
Combined effect of cross-plane acquisition (figure 6) and $H^1$ regularization (figure 5) for all inclusion types in figure 3, using six pushes under the cross-plane configuration (figure 4(b)). Reconstructions employ the full objective $J_3(p)$ (13) with regularization weight $\alpha = 10^{-3}$. (a) Bimodal Gaussian, (b) sharp-contrast, and (c) uncorrelated inclusions. Columns show ground truth, no noise, 30 dB, and 15 dB SNR; rows show shear modulus (top) and viscosity (bottom).

Figure 7 presents the final reconstructions obtained using the full objective $J_3(p)$ (13), which incorporates both the cross-plane acquisition strategy and the $H^1$ regularization term. Results are shown for all inclusion types from figure 3, reconstructed under the cross-plane configuration of figure 4(b) with six ARF pushes and regularization weight $\alpha = 10^{-3}$.

These results combine the improvements observed individually in figure 6 (cross-plane sampling) and figure 5 ($H^1$ regularization). The bimodal Gaussian case demonstrates enhanced contrast recovery and reduced noise sensitivity; the sharp-contrast inclusion shows sharper boundary preservation; and the uncorrelated case confirms the ability to separate elasticity and viscosity distributions. Overall, the combination of multi-plane data and smoothness regularization yields the most stable and physically consistent reconstructions across all inclusion types.

### 3.4. Discussion

The results demonstrate that combining cross-plane acquisition with $H^1$ regularization substantially improves the accuracy and robustness of viscoelastic reconstructions compared to conventional single-plane SWE. Cross-plane sampling (figures 4(b) and 6) enhances sensitivity to viscous dissipation by capturing energy loss across measurement planes, thereby reducing ambiguity in $\eta$ reconstructions. In contrast, $H^1$ regularization (figure 5) suppresses oscillatory artifacts and stabilizes the inversion under noisy conditions, though it also introduces mild ‘bleeding’ in viscosity where inclusion boundaries appear slightly diffused due to enforced smoothness. This trade-off is consistent with the gradient penalization effect of the $H^1$ term and persists in the combined results (figure 7), reflecting a balance between stability and spatial resolution.

The six-push configuration used here extends the multi-push framework of the prior correlation-based FWI study (Elmeliegy and Guddati 2024), in which up to four pushes were used. However, other push configurations were also tested and their effects observed. Generally we found that as we decreased the push count from 6 to 1, cross-plane reconstruction quality degraded for both parameters: RMS error increased from 3.7% to 10.5% for viscosity and 1.3% to 3.6% for elasticity at no noise. The effect was minimized as noise level increased but followed the same trend. Push separation for 3 pushes was tested; widening from 2 mm to 10 mm reduced viscosity RMS error from 9.0% to 6.8% and elasticity RMS error from 3.5% to 1.8%. Therefore, it is observed that the reconstruction quality using the cross-plane method generally benefits from greater push count and wider separation, with effects more prominent at lower noise levels.

Elasticity maps remain robust across all conditions, as the correlation-based objective is effective in matching phase information, thus capturing wave kinematics. The improvements in viscosity reconstructions underscore the complementary roles of enhanced acquisition and smoothness regularization. While the present study is limited to idealized synthetic data with reduced dimensionality, the observed gains highlight the potential of this framework to enable practical, noise-tolerant 3D viscoelastic imaging through multi-plane SWE acquisitions.

Practical considerations for clinical translation include both acquisition hardware and computational cost. The acquisition envisioned here uses multiple synchronized linear (1D) ultrasound arrays in a fixed holder, each recording one measurement plane simultaneously from a single ARF push. 2D matrix-array probes (Smith *et al* 1991, Gennisson *et al* 2015) and row-column addressed arrays (Seo and Yen 2009, Flesch *et al* 2017) are possible alternatives, though less aligned with this measurement geometry. Computationally, conventional time-of-flight SWE operates at clinical frame rates (Sigrist *et al* 2017), whereas FWI is several orders of magnitude slower owing to repeated forward solves and gradient evaluations. By way of reference, the inversions reported here completed in approximately 40 min per case on a 2020 MacBook Air (M1, 8 GB RAM). Bridging the remaining gap to clinical real-time imaging will require substantial further speedup; as possible avenues, GPU-accelerated FWI implementations (Yang *et al* 2015, Fabien-Ouellet *et al* 2017) and machine-learning surrogate or warm-start models for FWI (Araya-Polo *et al* 2018, Wu and Lin 2019) have reduced reconstruction times in geophysics by one to two orders of magnitude, with analogous gains directly applicable here. The present study targets methodological feasibility rather than clinical throughput, but these directions suggest FWI-based viscoelastic imaging can reach interactive speeds as the framework matures.

## 4. Conclusions

This study demonstrates the feasibility of reconstructing out-of-plane viscoelasticity images using regularized FWI for SWE with cross-plane acquisition strategy. By extending conventional 2D acquisitions to include multiple measurement planes per ARF push, the proposed approach captures dissipative behavior that would otherwise be poorly captured within a single plane. This additional cross-plane information enables improved conditioning of the inversion, particularly for viscosity, where dissipation is captured along paths that extend beyond the measurement plane. Combined with $H^1$ regularization, the framework produces stable and physically consistent reconstructions that recover both elasticity and viscosity fields under realistic noise conditions.

While the formulation here is limited to an idealized 2D setting, it isolates the essential physics of out-of-plane imaging and establishes a pathway toward volumetric reconstruction from standard 2D measurements. Future work will generalize this approach to fully 3D geometries, incorporate experimentally measured data, and explore adaptive strategies for plane selection and regularization strength. These developments will advance SWE toward quantitative 3D viscoelastic imaging, allowing true volumetric mapping of both stiffness and dissipation in heterogeneous biological tissues.

## Data Availability

The data used in this study are synthetically generated from numerical simulations. The simulation parameters and methodology are fully described in the manuscript, enabling reproducibility. The synthetic datasets are available from the authors upon reasonable request.
